# Hyperglycemia After Renal Transplantation: Frequency and Risk Factors

**DOI:** 10.5812/numonthly.10773

**Published:** 2013-03-30

**Authors:** Nahid Khalili, Zohreh Rostami, Ebrahim Kalantar, Behzad Einollahi

**Affiliations:** 1 Nephrology and Urology Research Center, Baqiyatallah University of Medical Sciences, Tehran, IR Iran; 2 Department of Immunology, Tehran University of Medical Sciences, Tehran, IR Iran

**Keywords:** Kidney Transplantation, Hyperglycemia, Diabetes Mellitus

## Abstract

**Background:**

Chronic renal failure is an important and common complication of diabetes mellitus; hence, renal transplantation is a frequent and the acceptable treatment in patients with diabetic nephropathy requiring renal replacement therapy. On the other hand, renal transplantation and its conventional treatment can lead to increased diabetes outbreak in normoglycemic recipients. Also, uncontrolled hyperglycemia may be increased and allograft lost thus decreasing patient survival.

**Objectives:**

We aimed to assess the frequency of hyperglycemia in transplant patients and its risk factors.

**Patients and Methods:**

A large retrospective study was performed on 3342 adult kidney transplant recipients between 2008 and 2010. Demographic and laboratory data were gathered for each patient. All tests were done in a single laboratory and hyperglycemia was defined as a fasting plasma glucose of > 125 mg/dL. Univariate and multivariate logistic regression analyses were used to determine the risk factors of hyperglycemia following kidney transplantation.

**Results:**

There were 2120 (63.4%) males and 1212 (36.3%) females. Prevalence of hyperglycemia was 22.5%. By univariate linear regression, hyperglycemia was significantly higher in patients with CMV infection (P = 0.001), elevated serum creatinine (P = 0.000), low HDL (P = 0.01), and increased blood levels of cyclosporine (P = 0.000). After adjusting for covariates by multivariate logistic regression, the hyperglycemia rate was significantly higher for patients with Cyclosporine trough level > 250 (P = 0.000), serum creatinine > 1.5 (P = 0.000) and HDL < 45 (P = 0.03).

**Conclusions:**

This study indicated that hyperglycemia is a common metabolic disorder in Iranian kidney transplant patients. Risk factors for hyperglycemia were higher Cyclosporine level, impaired renal function, and reduced HDL value.

## 1. Background

Diabetes mellitus (DM) is considered as one of the most costly diseases and important causes of end-stage renal disease (ESRD) throughout the world (1-5). Hyperglycemia is also a common complication among transplant patients without a history of DM. Although the new potent immunosuppressant agents have improved short-term and long-term outcomes after transplantation, these drugs can cause greater prevalence of hyperglycemia (6). In addition, DM may increase the risk of cardiovascular disease, infection, nephropathy, neuropathy and retinopathy (7).

Although some studies have shown that kidney transplant recipients with DM have an increased risk of allograft rejection (8-10), many studies indicated the similar result for patient and graft survival rates in diabetic transplant patients with good control of blood glucose level as compared to general transplant recipients without DM (3, 11-13).

## 2. Objectives

There are limited data available regarding the prevalence of hyperglycemia after kidney transplantation among Iranian transplant recipients (3, 14-16). Therefore, we aimed to evaluate the frequency of hyperglycemia in renal transplant patients and it’s risk factors in a large renal transplanted population in Iran.

## 3. Patients and Methods

### 3.1. Patient Population

We carried out a retrospective analysis lab data of all adult patients (age > 18 years) who underwent renal transplantation at 8 academic hospitals of Tehran, Iran referred to Gholhak laboratory during 2008-2010. Living related and deceased kidney transplants were both included. We obtained 14986 lab data in 3342 renal transplant recipients within the period of the study. Ethics approval was supplied by the local ethics committee of Baqiyatallah university. We excluded patients who suffered from transient hyperglycemia due to steroid pulse, incomplete data and rejected allograft.

### 3.2. Data Collection

Data recorded for all of the patients were age, sex, fasting plasma glucose level (FBS), serum creatinine concentration (Cr), low-density lipoprotein (LDL), high-density lipoprotein (HDL), uric acid, hemoglobin (Hb), trough level of cyclosporine (C0) and cytomegalovirus Ag (CMV Ag).

### 3.3. Hyperglycemia Definition

We defined hyperglycemia as fasting plasma glucose of > 125 mg/dL, according to the world American diabetes association (17).

### 3.4. Immunosuppressive Regimen

The maintenance of immunosuppression in all patients was based on Cyclosporine neoral plus Mycophenolate mofetil or Azathioprine and Prednisolone. The amount of cyclosporine given to transplant patients was mostly based on drug levels in the blood. Monitoring of Cyclosporine using its trough levels was periodically performed at different times and dose was adjusted as necessary. In our treatment strategy, target therapeutic ranges for Cyclosporine levels were 200 to 300 ng/mL during the first 3 months, 100 to 250 ng/mL during 4 to 12 months and 100 to 150 ng/mL past 1 year from transplantation.

### 3.5. Statistical Analysis

CMV pp65 antigenemia was detected by Brite TM Turbu (IQ Products, CMV Groningen, Netherlands) and Cyclosporine levels were determined with Cobas Mira-Plus analyzer (Roche). Statistical analyses were performed using the SPSS software (statistical package for the social sciences, version 17.0, SPSS Inc, Chicago, III, USA). All numeric data were presented as mean ± standard deviation. Differences between the quantitative variables were compared using the Chi square test or the Fisher exact test. The student t test was used for evaluating continuous quantitative variables. The univariate linear regression and multivariate logistic regression were performed for risk factors associated with hyperglycemia. A P value of less than 0.05 was considered statistically significant.

## 4. Results

### 4.1. Demographics

There were 3342 renal transplant patients, 2120 males (63.4%) and 1212 females (36.3%), referred to a single laboratory from 8 academic transplant centers. The majority of the patients (93.4%) received a kidney from a living donor (85.2% unrelated and 8.2% related). The mean age of recipients was 37 ± 16 years (range, 18-79 years). The majority of patients were followed up for more than one year (92.6%), while 3.5% of the recipients had 3-12 month follow ups and 3.9% had less than 3 months of follow ups. Patients with hyperglycemia were older compared to normoglycemic recipients (Table 1). The prevalence of hyperglycemia was 22.5% of the cases.

**Table 1.** Patient Characteristics and Laboratory Data in Hyperglycemic and Normoglycemic Kidney Transplant Recipients

Variable	Normoglycemic Group	Hyperglycemic Group	P value
**Age of recipient, y, mean ± SD**	35.8 ± 15.6	46.9 ± 12.0	0.000
**Recipient sex, Male/Female, %**	62.8/37.2	66.4/33.6	0.37
**CMV Ag, Positive/Negative, %**	4.7/95.3	11.7/88.3	0.000
**Trough level of Cyclosporine, ng/mL, mean ± SD**	182 ± 121	230 ± 150	0.000
**Creatinine, mg/dL, mean ± SD**	1.54 ± 0.97	1.79 ± 1.20	0.000
**HDL-Cholesterol, mg/dL, mean ± SD**	49 ± 15	47 ± 17	0.15
**LDL-Cholesterol, mg/dL, mean ± SD**	101 ± 35	105 ± 36	0.74
**Hemoglobin, g/dL, mean ± SD**	12.5 ± 2.2	12.1 ± 2.3	0.31

### 4.2. Risk Factors of Hyperglycemia After Transplantation

Hyperglycemia patients had significantly higher rates of CMV infection, elevated plasma cyclosporine levels and increased plasma creatinine levels as compared to normoglycemic recipients Table 1, 2. Although hyperglycemia patients had higher LDL, this difference was not statistically significant (P = 0.7). Despite of the non significant correlation which was observed between hyperglycemia and Hb or HDL concentrations as quantitative values, these biochemical markers as qualitative entities had a significant relationship Table 2.

By univariate linear regression, the prevalence of hyperglycemia was significantly higher in patient with CMV infection (P = 0.001), impaired renal allograft function (P = 0.000), low HDL (P = 0.01), and elevated plasma cyclosporine levels (P = 0.000). After adjusting for covariates by multivariate logistic regression, hyperglycemia rate was significantly higher for patients with plasma cyclosporine trough level > 250 (CI = 0.27-0.98, P = 0.000), plasma Cr > 1.5 mg/dL (CI = 0.93-0.98, P = 0.000) and HDL < 45 mg/dL (CI = 0.95-0.99, P = 0.03).

**Table 2.** Significant Qualitative Laboratory Data in Hyperglycemic and Normoglycemic Kidney Transplant Recipients

Variable		Overall, %	Normoglycemic Group, %	Hyperglycemic Group, %	P value
**Trough level of Cyclosporine, ng/mL**	** **				0.000
** **	**> 250**	24.4	21.2	35.3	
** **	**≤ 250**	75.6	78.8	64.7	
**Creatinine, mg/dL**	** **				0.000
** **	**> 1.5**	37.9	35.8	45	
** **	**≤ 1.5**	62.1	64.2	55	
**HDL-Cholesterol, mg/dL**	** **				0.02
** **	**> 45**	47.5	52.1	46	
** **	**≤ 45**	52.5	47.9	54	
**Hemoglobin, **	** **				0.01
**g/dL**	**> 11**	26.7	32.2	25.1	
** **	**≤ 11**	73.3	67.8	74.9	

## 5. Discussion

In the current study, hyperglycemia was a frequent problem among renal transplant recipients. Perez-Flores et al. showed that hyperglycemia was a common complication after kidney transplantation and it was present in 65% of their patients (18). The incidence of post-transplant diabetes mellitus ranges from 2% to 50% of cases in different series, reflecting the wide variation in the definition of the disorder, population, and immunosuppressive regimen (19).

Hyperglycemia is a common and contributing factor for the outcome of kidney transplantation. Our study was able to confirm previous findings by showing an increased risk of renal allograft impairment associated with PTDM (8, 20, 21). Unless adequately controlled, DM after transplantation enhances the risk of renal graft loss (21).

Several risk factors for the development of hyperglycemia after transplantation have been previously described, which include advanced age, male donor, infections (HCV, EBV, CMV), obesity (13), immunosuppressive agents (particularly Prednisolone and Cyclosporine) and dyslipidemia (10). In our study, high blood level of cyclosporine seemed to be an important factor for the development of altered glucose metabolism. We also demonstrated that our hyperglycemic patients had significantly higher rates of CMV infection as compared to normoglycemic cases; this matches reports from previous studies (22, 23). Conversely, two small studies showed that there was no significant correlation between DM and CMV infection after renal transplantation (24, 25). We were also able to demonstrate a significant increase in the risk of hyperglycemia with advanced age, which is consistent with other studies (25-29).

Although no significant correlation was seen between hyperglycemia and Hb concentration, hyperglycemic recipients were more likely to be anemic as compared to normoglycemic patients; also matching other research (27).

The main limitation of the present study resides in its retrospective design. As a result, data about family history, BMI, metabolic syndrome, and BP were not accessible. However; we believe that this limitation exists in nearly all retrospective studies using secondary data collection. We could not consider all confounding factors in this study.

In conclusion, this study indicated that hyperglycemia is a common metabolic disorder in Iranian kidney transplant patients. These results confirmed the importance of an appropriate control of cyclosporine levels among patients undergoing kidney transplantation.
